# Degradation and Damage Effects in GaN HEMTs Induced by Low-Duty-Cycle High-Power Microwave Pulses

**DOI:** 10.3390/mi16101137

**Published:** 2025-10-01

**Authors:** Dong Xing, Hongxia Liu, Mengwei Su, Xingjun Liu, Chang Liu

**Affiliations:** 1 Key Laboratory for Wide Band Gap Semiconductor Materials and Devices of Education Ministry, School of Microelectronics, Xidian University, Xi’an 710071, China; xd_6268@163.com (D.X.); smw6201@163.com (M.S.); liuxj_icer@163.com (X.L.); xd_liuchang@163.com (C.L.); 2 Science and Technology on Reliability Physics and Application Technology of Electronic Component Laboratory, China Electronic Product Relibility and Environmental Testing Research Institute, Guangzhou 511370, China

**Keywords:** Gallium nitride (GaN), high electron mobility transistors (HEMTs), high-power microwave (HPM), degradation, damage effect, trap

## Abstract

This study investigates the effects and mechanisms of high-power microwave on GaN HEMTs. By injecting high-power microwave from the gate into the device and employing techniques such as DC characteristics, gate-lag effect analysis, low-frequency noise measurement, and focused ion beam (FIB) cross-sectional inspection, a systematic investigation was conducted on GaN HEMT degradation and failure behaviors under conditions of a low duty cycle and narrow pulse width. Experimental results indicate that under relatively low-power HPM stress, GaN HEMT exhibits only a slight threshold voltage shift and a modest increase in transconductance, attributed to the passivation of donor-like defects near the gate. However, when the injected power exceeds 43 dBm, the electric field beneath the gate triggers avalanche breakdown, forming a leakage path and causing localized heat accumulation, which ultimately leads to permanent device failure. This study reveals the physical failure mechanisms of GaN HEMTs under low-duty-cycle HPM stress and provides important guidance for the reliability design and hardening protection of RF devices.

## 1. Introduction

Gallium nitride (GaN) high-electron-mobility transistors (HEMTs), featuring high breakdown field strength, high channel carrier density, and high drift velocity, are representative wide-bandgap power semiconductor devices that have been widely applied in power electronics and radio frequency fields [1,2,3]. With the increase in power density, GaN HEMT devices also need to deal with increasingly complex electromagnetic environments such as high-power microwaves (HPMs), high-power electromagnetic pulses (EMPs), and particle irradiation. Reliability problems have become one of the biggest obstacles to market competitiveness. HPM pulses can be coupled into the radio frequency (RF) front end through the front door (antenna) and the back door (microstrip line or power line) [4,5]. As a key component of the RF front-end receiving chain, the foremost HEMT is the part most susceptible to damage. Therefore, it is necessary to investigate the damage effects of HPM on GaN HEMT devices [6,7,8].

The failure mechanisms of HEMTs have been studied by many researchers. Y. M. Zhang et al. [9] analyzed the thermal response of GaN HEMTs subjected to electrical pulses with different duty cycles and frequencies, concluding that a low duty cycle combined with high-frequency operation is favorable for prolonging the device lifetime and improving reliability. L. Zhou et al. [10] investigated the damage caused by thermal stress in GaN HEMTs subjected to HPM pulses, and the results indicated that the field plate near the gate is a vulnerable region. Zhang Y et al. [11] systematically investigated the impact of thermal effects and inverse piezoelectric effects on the performance and reliability of GaN HEMTs using TCAD simulations. Qin Y et al. [12] explored the distinct influences of self-heating effects and HPM effects on enhancement-mode p-gate AlGaN/GaN HEMTs through both simulation and experimental verification. Li X et al. [13] analyzed the transient thermal behavior and surface electric field characteristics of GaN HEMTs subjected to HPM stress across various power levels and revealed that device failure results from thermo-electrical multi-physics coupling rather than simple thermal burnout. Above all, research on the failure mechanisms of GaN HEMTs under HPM pulses has primarily focused on heat accumulation at relatively high pulse repetition frequencies (PRFs) and electrical breakdown at lower PRFs. However, under extremely low-duty-cycle conditions, the degradation and failure processes of the device may involve distinct physical mechanisms, such as localized defect responses, transient electric field concentration, and non-equilibrium carrier dynamics. Therefore, a systematic investigation of the damage mechanisms under these conditions not only helps to elucidate the failure boundaries of GaN HEMTs in extreme electromagnetic environments but also provides essential theoretical and experimental foundations for reliability design and hardening of RF devices.

In this study, GaN HEMTs were subjected to high-power microwave pulses applied via the gate, leading to the identification of a failure mode distinct from those previously reported. To investigate this degradation and failure mode, multiple failure analysis techniques were employed in combination, including gate-lag, low-frequency noise (LFN), focused ion beam (FIB), and scanning electron microscopy (SEM), to perform failure localization and microscopic damage morphology characterization, thereby determining the damage mechanism of the devices.

## 2. Structure and Measurement Systems of GaN HEMTs

**Figure 1:**
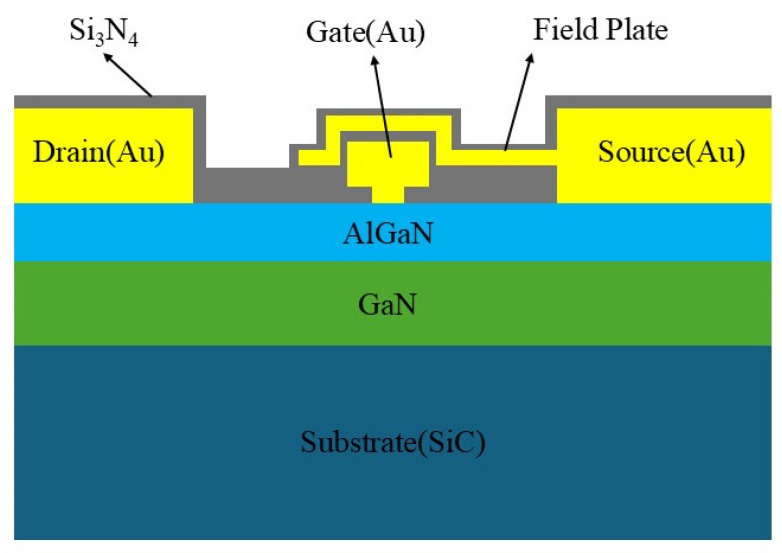
Structure of the GaN HEMT.

We selected a batch of GaN HEMTs with identical structures, the cross-sectional schematic of which is shown in Figure 1. The device is fabricated on a SiC substrate, followed by the sequential growth of a GaN buffer layer, an AlGaN barrier layer, and a passivation layer. A two-dimensional electron gas (2DEG) is formed at the AlGaN/GaN heterojunction interface. The gate is a Schottky contact, while the source and drain are Ohmic contacts. The field plate located near the gate is tied to the source terminal, redirecting the feedback capacitance from the gate–drain path to the gate–source path, which reduces the device gain drop. All gold-based metal electrodes are covered by a silicon nitride (Si_3_N_4_) thin film. The specific structural parameters of the device are listed in Table 1. In this work, the drain voltage was set at 10 V, and the gate voltage was set at –2.3 V, corresponding to a typical drain current of 100 mA. The typical operating frequency range is 2–6 GHz, with a small-signal gain of approximately 24 dB and a noise figure as low as 1.5 dB. To ensure statistical reliability, ten devices from the same batch with excellent initial parameter consistency were selected for reliability testing. All tested devices exhibited similar degradation and failure phenomena. From the resulting dataset, the device whose performance was closest to the median was chosen for detailed analysis.

**Figure 2:**
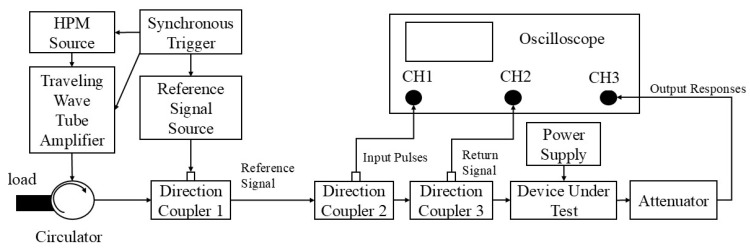
Diagram of the measurement system.

Figure 2 presents the schematic diagram of the measurement system used to investigate the failure mechanisms of GaN HEMTs under HPM pulse injection. The measurement system consists of an HPM signal source, a driver amplifier, a circulator, a power supply, the HEMT under test, a directional coupler, an attenuator, a vector network analyzer, an oscilloscope, and a power meter. Experimental methods for investigating high-power microwave (HPM) effects generally include irradiation and injection approaches [14]. In this study, the injection method was employed, in which microwave pulses were directly injected into the HEMT through a transmission cable. During the measurement process, a series of pulses generated by the HPM source were gradually amplified by a solid-state power amplifier, then injected into the device under test (DUT) via a circulator and cascaded directional couplers. The output signals were subsequently routed through a directional coupler and an attenuator into a spectrum analyzer to monitor the output in the frequency domain. The coupled ports of directional couplers 1, 2, and 3 were used to monitor the injected pulse power threshold, reflected signals, and output signal waveforms, respectively. Attenuators were employed to protect the oscilloscope.

## 3. Results and Discussion

### 3.1. Measurement of Device Burnout Peak Power

**Figure 3:**
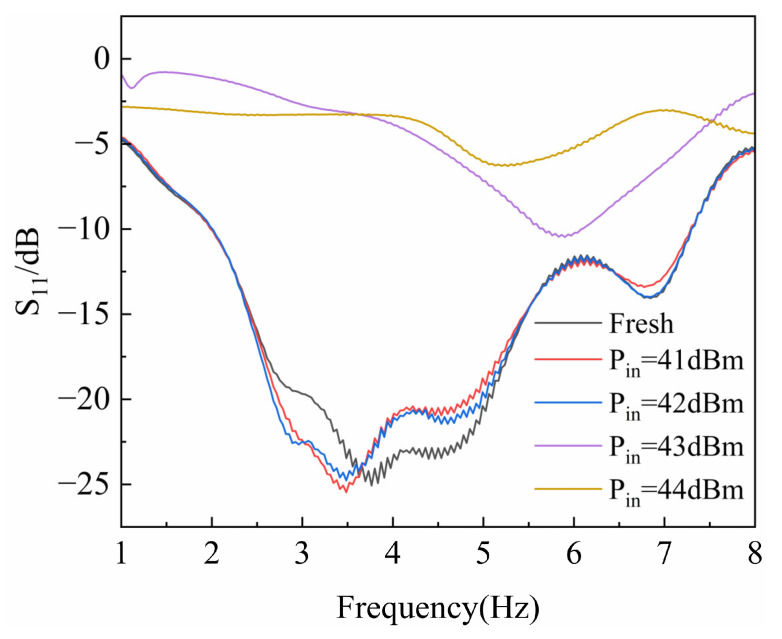
Variation in the GaN HEMT S_11_ parameter with injected power.

**Figure 4:**
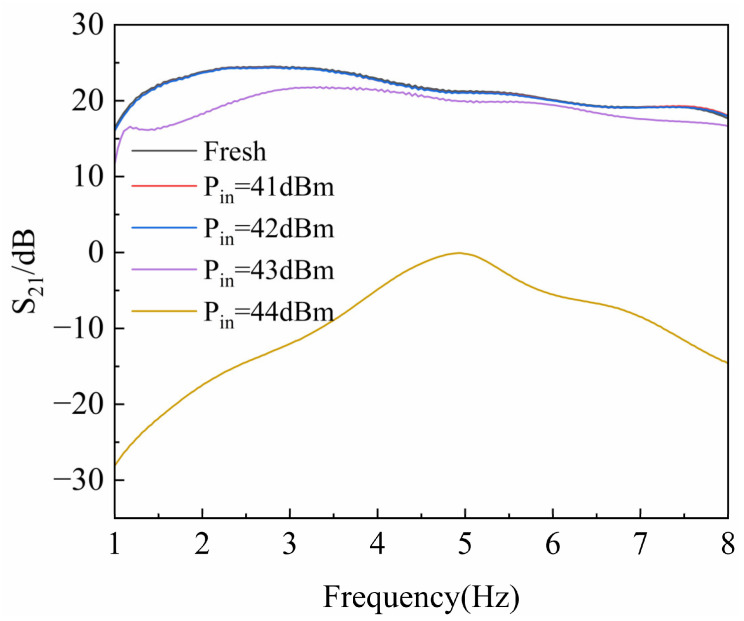
Variation in the GaN HEMT S_21_ parameter with injected power.

The preliminary tests were conducted to measure the RF characteristics of the GaN HEMT devices. During the initial stage, high-power microwave pulses with power levels of 41 dBm, 42 dBm, 43 dBm, and 44 dBm were injected. The HPM pulses had a frequency of 3.5 GHz, a pulse width of 30 ns, rise and fall times of 18 ns, a period of 2 ms, and a total of 200 pulse cycles. The gate voltage was set to −2.3 V, and the drain voltage was at 10 V. Figure 3 and Figure 4 show variation in the device reflection coefficient (S_11_) and gain coefficient (S_21_) as a function of input power. As observed in Figure 3, the reflection coefficient of the GaN HEMT exhibits an abrupt change around 43 dBm, and Figure 4 shows that the gain of the device also undergoes a sudden shift at this point. Therefore, a power level 0.5 dBm below the damage threshold was selected to ensure that the device would not fail instantaneously.

### 3.2. Effect of HPM Stress on DC Characteristics

**Figure 5:**
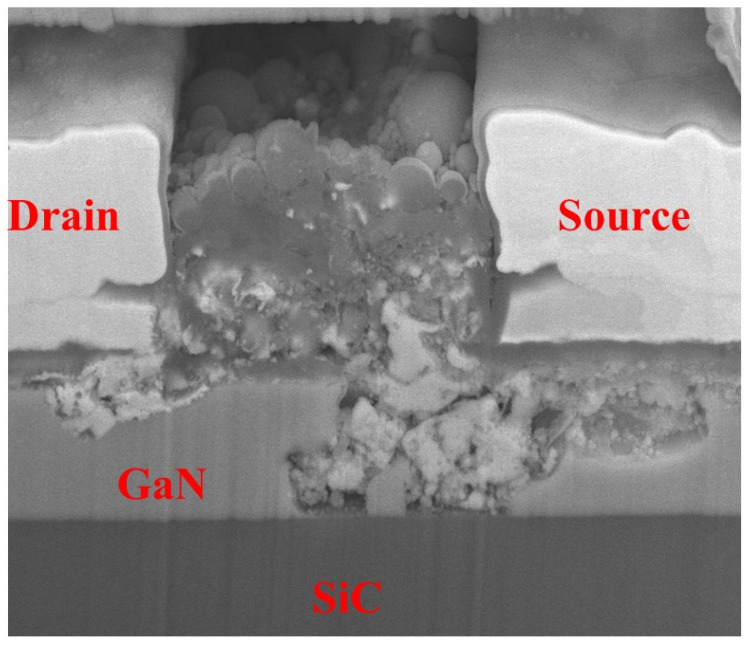
Damage morphology of the GaN HEMT after testing.

The DUT was subjected to HPM pulses with an injection power of 42.5 dBm, a frequency of 3.5 GHz, a pulse width of 66 ns, and a period of 2 ms, with the gate voltage set to −2.3 V,  and the source–drain voltage set to 10 V. After sequentially injecting 10, 20, 50, 80, 100, 200, 500, and 800 pulses, the device remained intact. When the injection power was increased to 43 dBm, the device failed after 10 pulses. To characterize the failure, the damaged region was analyzed using FIB cross-sectioning. The morphology of the burnout cross-section is shown in Figure 5, from which severe damage to the gate can be observed. The preliminary assumption is that the failure mechanism may be attributed to defect accumulation during the first 800 pulses and/or sudden breakdown under the 43 dBm stress [15].

**Figure 6:**
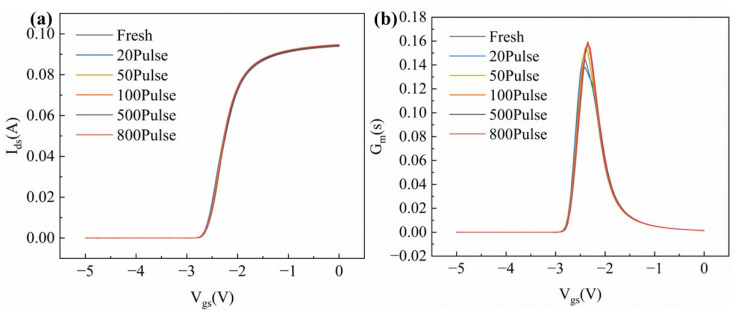
Electrical performances after varying numbers of pulses: (**a**) the transfer characteristics; (**b**) the transconductance characteristics.

To reveal the impact of HPM pulses on the DC characteristics of GaN HEMT devices, the transfer characteristics (I_ds_–V_gs_) and output characteristics (I_ds_–V_ds_) of the GaN HEMT were measured before and after the experiments. Figure 6a,b show the transfer characteristics and transconductance curves of the device before and after HPM stress, respectively. During the measurements, the drain voltage was set to a constant 0.5 V, and the gate voltage was swept from −5 V to 0 V in increments of 0.05 V. Both the transfer and transconductance (G_m_) curves exhibited slight rightward shifts, with the threshold voltage (Vth) drifting from −2.61 V to −2.56 V. This small shift does not significantly affect the electrical characteristics of the device. The G_m_ of the device initially increased with the cumulative number of injected pulses and then gradually saturated, indicating an enhancement of the gate control capability. The enhancement of gate control is generally attributed to the reduction in defect density near the gate [16].

**Figure 7:**
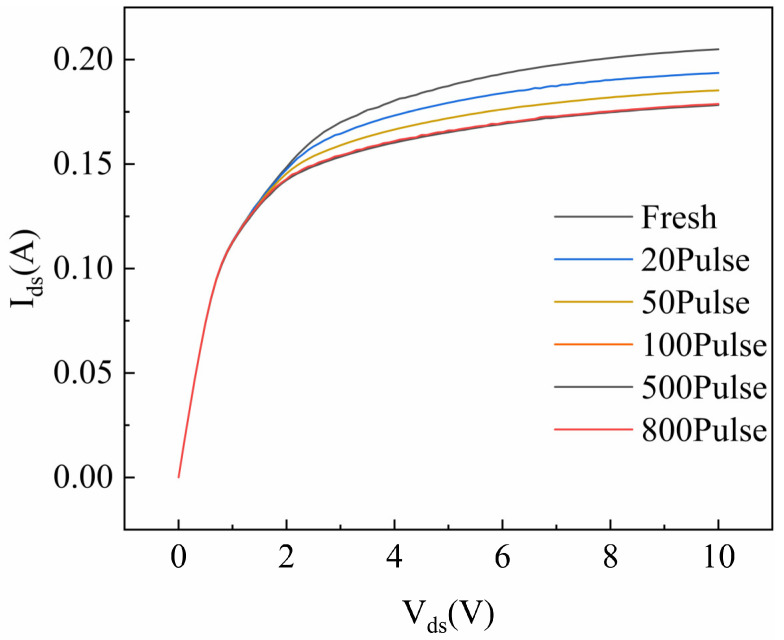
The output characteristics after varying numbers of pulses.

Figure 7 shows the output characteristics of the device before and after HPM pulse injection. The drain–source voltage ranged from 0 to 10 V, and the gate–source voltage ranged from −2 V to −3 V in steps of 0.25 V. The device’s saturation current exhibits a decreasing trend, dropping from 0.208 A to 0.179 A after HPM stress. However, once the cumulative number of injected pulses reaches 500, the saturation current density no longer decreases, indicating that further accumulation of pulses does not lead to additional degradation of the device’s electrical characteristics. The drain–source current collapse observed in fresh GaN HEMTs is primarily linked to the virtual gate effect, where a high drain bias near cutoff causes electrons to become trapped in a defect located at the AlGaN surface and within the barrier near the gate [17]. Studies have shown that positively charged centers generated by the ionization of donor-like defects near the gate are the primary cause of the virtual gate effect, which in turn leads to the degradation of the gate controllability of the device [18]. HPM injection may passivate some of these donor-like defects near the gate, and the reduction in defect concentration at the AlGaN surface near the gate leads to an increase in device transconductance [16]. The 2DEG concentration in GaN HEMTs is also closely related to the concentration of donor-like surface defects on AlGaN [18]. A reduction in these surface defects can indirectly lead to a decrease in the device’s saturation drain current density.

### 3.3. Effect of HPM Stress on Gate-Lag Characteristics

**Figure 8:**
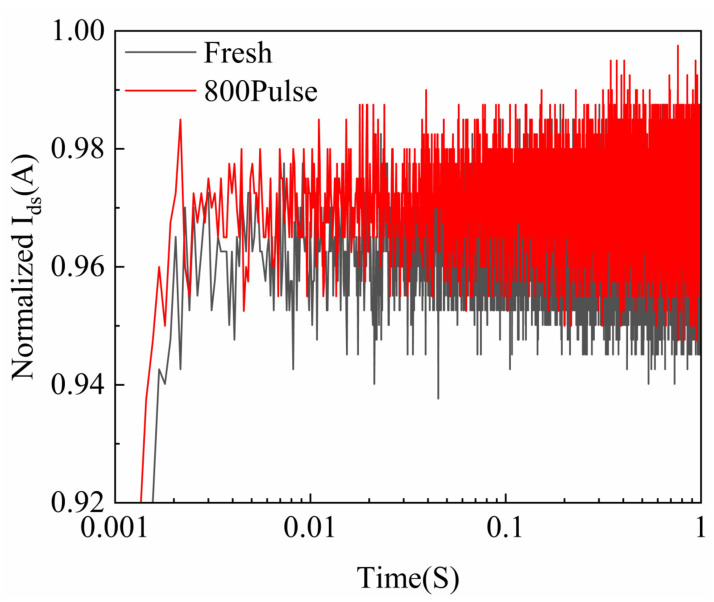
Gate-lag characteristics of GaN HEMTs before and after HPM stress.

The gate-lag characteristics are an important method for characterizing defects near the gate region of GaN HEMTs. Figure 8 shows the gate-lag curves of GaN HEMTs before and after HPM stress. The test conditions for these measurements are as follows: First, under the condition of Vgs = −5 V and Vds = 0 V, the device was maintained in the OFF state for 100 s. Subsequently, the gate voltage was switched to 0 V, thereby driving the device from the OFF state to the ON state. The drain current transient was recorded for 1 s after the gate bias was switched. For the fresh device, an electric field directed from the drain to the gate is formed as the negative voltage is applied to the gate, causing electrons to be trapped by original defects within the AlGaN layer or at the interface [19]. These occupied defects are typically situated near the drain, close to the gate, leading to depletion of the 2DEG in these regions. When the negative gate voltage is removed, the trapped electrons are not immediately released and require some time to recover [20]. After the device undergoes HPM stress, it reaches a steady state more quickly, indicating a reduction in defect concentration near the gate. This suggests that the accumulation of extremely low-duty-cycle HPM pulses does not lead to defect buildup that would cause device failure [21].

### 3.4. Effect of HPM Stress on Low-Frequency Noise

**Figure 9:**
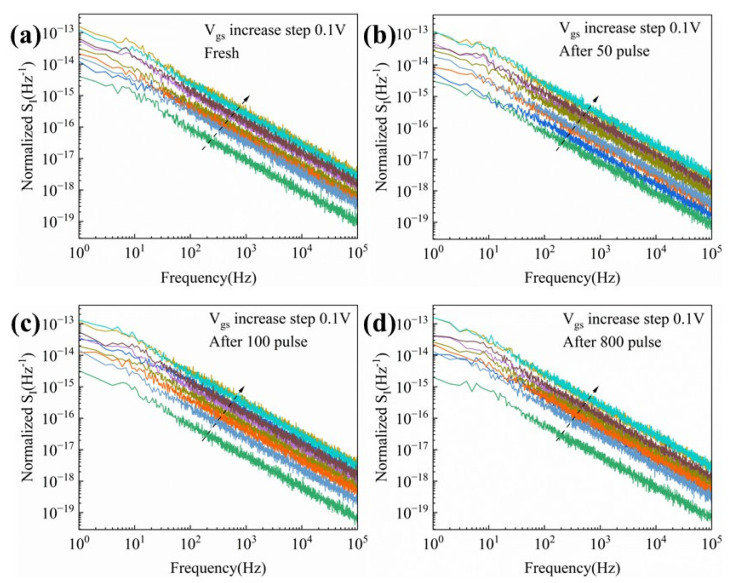
S_I_ versus frequency curves of the device before and after HPM stress application: (**a**) fresh device; (**b**) after 50 cumulative pulses; (**c**) after 100 cumulative pulses; (**d**) after 800 cumulative pulses.

LFN measurements have been demonstrated to be an effective technique for characterizing channel defects and interface state density. To further investigate the impact of HPM stress on GaN HEMTs, LFN spectra were measured under various gate bias conditions. The drain bias was fixed at 0.1 V, and the noise density (S_I_) was measured as a function of frequency while the gate voltage was swept from −2.8 V to −2 V in steps of 0.1 V. Figure 9a presents the original S_I_ versus frequency curve, while Figure 9b–d show S_I_ versus frequency after 50, 100, and 800 cumulative injected pulses, respectively.

**Figure 10:**
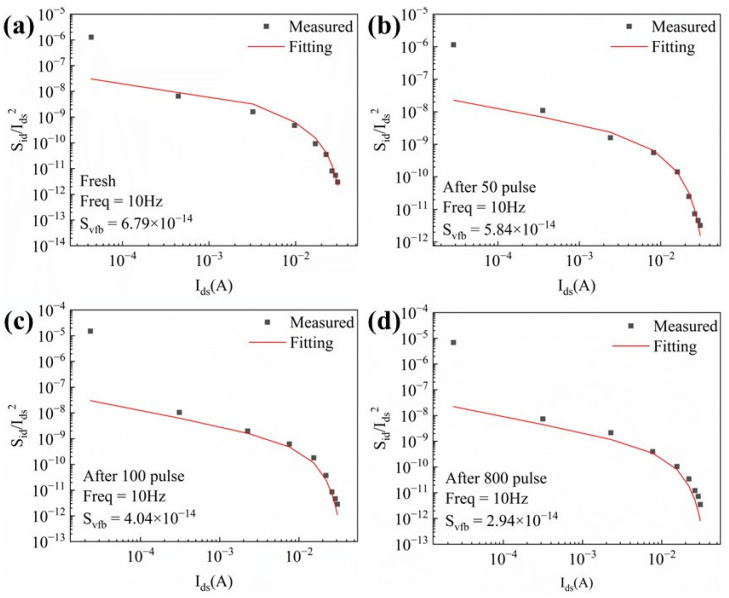
S_I_/I_d_^2^ at 10 Hz versus I_d_ for the fresh device and the devices after HPM stress application: (**a**) fresh device; (**b**) after 50 cumulative pulses; (**c**) after 100 cumulative pulses; (**d**) after 800 cumulative pulses.

The normalized S_I_/I_d_^2^ values versus the drain current for the fresh device, and after applying 50, 100, and 800 cycles of HPM stress, respectively, taken at 10 Hz, are shown in Figure 10. The origin of the channel 1/f noise can be explained using the carrier number fluctuation model, which describes the noise mechanism in detail by considering charge trapping and emission between interface traps and the channel [22]. S_I_/I_d_^2^ can be expressed by the following equation:

(1) $$\frac{S_{I}}{I^{2}}={\left(g_{m}/I\right)}^{2}S_{vfb}$$

S_vfb_ represents the reference noise spectral density and can be used as a fitting coefficient, whose primary role is to optimize the agreement between the experimental data points and the theoretical curve. Before pulse injection, the device exhibited an S_vfb_ of 6.79 × 10^−14^. After cumulative injections of 50, 100, and 800 pulses, S_vfb_ decreased to 5.84 × 10^−14^, 4.04 × 10^−14^, and 2.94 × 10^−14^. The trap density (N_t_) was calculated using the following equation:

(2) $$S_{vfb}=q^{2}kT\lambda N_{t}/WLfC_{b}^{2}$$

where q is the elementary charge, N_t_ is the volume trap density, T is the thermal energy, λ = 0.5 nm is the AlGaN/GaN conduction band alignment, L is the gate length, W is the gate width, C_b_ is the AlGaN unit barrier capacitance, and f is the frequency [22]. The model assumes that defects in the AlGaN barrier layer beneath the gate are uniformly distributed. In practice, however, defects may predominantly occur in a narrower barrier region near the gate edges. From (2), N_t_ can be extracted, and the results show that N_t_ is 1.55 × 10^15^ cm^−3^eV^−1^, 1.33 × 10^15^ cm^−3^eV^−1^, 9.21 × 10^14^ cm^−3^eV^−1^, and 6.70 × 10^14^ cm^−3^eV^−1^ for the fresh device and for the devices after applying 50, 100, and 800 cycles, respectively. Obviously, the density of traps near the gate was halved after the stress was applied. This further confirms that HPM signals with extremely low duty cycles do not cause cumulative defect generation that would otherwise lead to device failure.

### 3.5. Mechanism Analysis of Burnout Behavior of GAN HEMT

**Figure 11:**
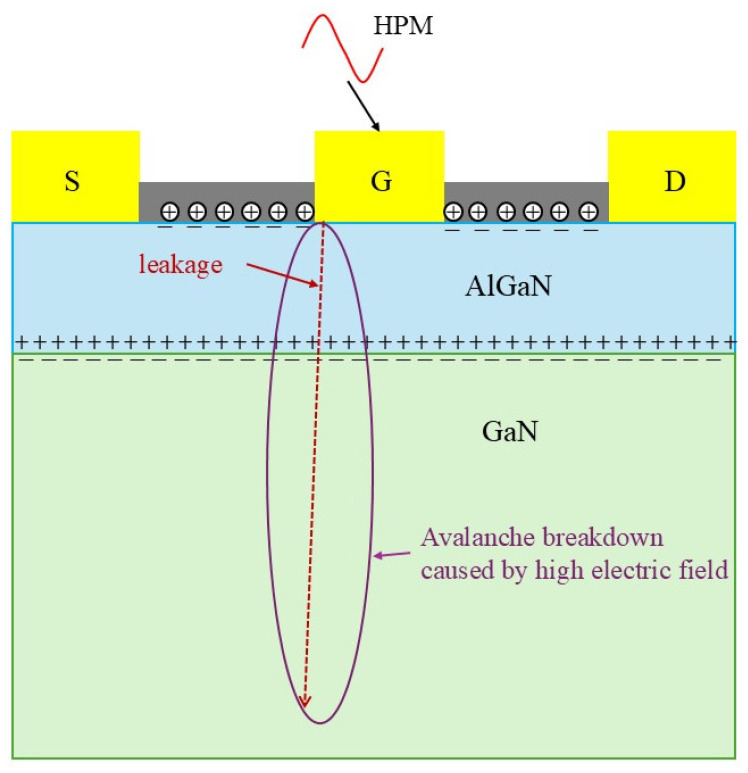
Schematic diagram of the damage process of GaN HEMTs under HPM stress.

Based on the above experimental results, Figure 11 illustrates the physical degradation and damage processes of GaN HEMT under HPM exposure. For fresh GaN HEMTs, traps are present in both the AlGaN barrier layer and on the AlGaN surface [23,24]. Previous studies indicate that the failure mechanisms of GaN HEMTs exposed to HPM stress can generally be divided into three categories: (1) when the pulse duration td is extremely short (td < 10 ns), electrical breakdown occurs without sufficient time for heat generation; (2) for the pulses with large value td (10 ns < td < 100 μs), electrothermal failure always occurs; (3) when the duration is very long (td > 100 μs), the device reaches a steady state where thermal equilibrium is established, occasionally accompanied by hot-carrier injection [25]. In this experiment, the HPM pulse td was 66 ns, and electrothermal failure occurred. When HPM pulses are injected into the device through the gate, an extremely high electric field is generated at the gate edge near the drain [26,27]. If this electric field does not reach the breakdown strength of the material near the gate, HPM stress can passivate donor-like surface defects on the AlGaN near the gate, resulting in an increase in the device gate transconductance [16]. The reduction in donor-like defect concentration on the AlGaN surface near the gate decreases the positive charge generated by ionization, depletes the 2DEG in the channel, and consequently reduces the device saturation drain current [18]. As the injected pulse power increases, when the electric field near the gate exceeds the breakdown strength of the GaN material, avalanche breakdown occurs between electrons and holes in GaN [28]. Moreover, in GaN HEMTs, substrate–buffer lattice mismatch introduces a large number of material defects [29], which contributes to the establishment of leakage conduction paths. When the buffer layer leakage becomes excessive, the device may break down in advance, which explains the burn morphology observed. After the HPM stress disappears, the drain bias of the device remains, with the drain voltage still at 10 V. Thus, the leakage pathway continues to exist, eventually leading to the total device being completely burned. This explains why HPM stress can fully burn out the GaN HEMTs.

## 4. Conclusions

In conclusion, we investigate the degradation and damage effects and underlying mechanisms of GaN HEMTs under high-power microwave exposure. It was discovered that for low-duty-cycle, narrow-width HPM pulses, relatively low input power only causes slight degradation of the device’s electrical performance without inducing failure. Only when the input power is sufficiently high does the strong electric field near the gate induce avalanche breakdown near the gate, forming a leakage path. Post-HPM stress, the device’s own bias exploits the leakage path, expanding the burnout region to complete failure, highlighting considerations for RF device protection and high-power microwave use.

## Figures and Tables

**Table 1:** Main parameters of GaN HEMT.

Parameter	Data
Gata length	0.25 μm
Source length	28 μm
Drain length	28 μm
AlGaN thickness	22 μm
GaN thickness	1.7 μm
SiC thickness	100 μm
Drain–gate length	2.65 μm
Gate–source length	0.8 μm

## Data Availability

The original contributions presented in the study are included in the article; further inquiries can be directed to the corresponding author.
